# Long Short-Term Memory Networks’ Application on Typhoon Wave Prediction for the Western Coast of Taiwan

**DOI:** 10.3390/s24134305

**Published:** 2024-07-02

**Authors:** Wei-Ting Chao, Ting-Jung Kuo

**Affiliations:** 1 Department of Applied Artificial Intelligence, Ming Chuan University, Taoyuan 33348, Taiwan; wtchao@mail.mcu.edu.tw; 2 Center of Excellence for Ocean Engineering, National Taiwan Ocean University, Keelung 20224, Taiwan

**Keywords:** IoUT, typhoon waves, typhoon parameters, Long Short-Term Memory, long lead time prediction

## Abstract

Huge waves caused by typhoons often induce severe disasters along coastal areas, making the effective prediction of typhoon-induced waves a crucial research issue for researchers. In recent years, the development of the Internet of Underwater Things (IoUT) has rapidly increased the prediction of oceanic environmental disasters. Past studies have utilized meteorological data and feedforward neural networks (e.g., BPNN) with static network structures to establish short lead time (e.g., 1 h) typhoon wave prediction models for the coast of Taiwan. However, sufficient lead time for prediction remains essential for preparedness, early warning, and response to minimize the loss of lives and properties during typhoons. The aim of this research is to construct a novel long lead time typhoon-induced wave prediction model using Long Short-Term Memory (LSTM), which incorporates a dynamic network structure. LSTM can capture long-term information through its recurrent structure and selectively retain necessary signals using memory gates. Compared to earlier studies, this method extends the prediction lead time and significantly improves the learning and generalization capability, thereby enhancing prediction accuracy markedly.

## 1. Introduction

The Internet of Things (IoT) is a network system composed of interconnected devices, including computing devices, machines, and digital mechanism equipment, without requiring additional interaction between humans or between humans and devices. IoT allows the exchange of information among IoT devices through cloud connectivity. IoT devices can often collect information via small single-board computers’ sensors and share the data after edge computing and data analysis [1]. The Internet of Underwater Things (IoUT) is a network structure composed of several interconnected sensors that can be used to achieve underwater detection, environmental monitoring, and oceanic disaster prediction [2].

Taiwan is located on the path of typhoons in the northwest Pacific Ocean and is affected by an average of three to four typhoons yearly. Long-period waves are generated as typhoons with strong winds and massive energy pass over the ocean surface. As typhoons approach land, the waves are shoaling due to the influence of the topography, and the wave height further increases. This process causes flooding in low-lying coastal areas and may damage seawalls and lead to coastal erosion, resulting in a loss of lives and properties. In recent years, global warming has intensified climate change, increasing the frequency and intensity of extreme weather events and raising the risk of typhoon disasters [3,4,5]. Collecting typhoon information and developing rapid analysis and prediction tools have become important challenges in coastal engineering protection [6,7].

In general, typhoon wave prediction methods can be divided into the following three types: (1) empirical methods based on statistics regression or experience used for rapid initial predictions, such as the SMB method [8]; (2) hydrodynamic models based on physical principles, for example, the SWAN model [9,10] and WW3 model [11]; and (3) data-driven models (e.g., neural networks) [12,13,14]. Empirical methods utilize parameters related to typhoons, such as the maximum wind speed, forward speed, equivalent fetch length, and radius of the typhoon, to estimate the maximum significant wave height that may occur within the wind field [8,15,16]. Regression analysis can provide simple and rapid estimates of typhoon-induced waves. However, these methods can only roughly describe possible wave heights and periods. Furthermore, the formula needs to be adjusted in different water depth conditions, which also require empirical accumulation and judgment.

Hydrodynamic models constructed from a physical perspective are primarily used for broad-scale wave estimation, such as the Wave Analysis Model (WAM) based on the wave energy balance equation (WAMDI Group). Subsequent research incorporated physical evolution mechanisms into the balance equations to more comprehensively describe the changes in wave shoaling and dissipation near shorelines, resulting in the SWAN model [9,10]. Numerical models can provide more information, such as wave height, period, and direction at various depths. However, they require numerous input parameters, and adjustments to relevant parameters are necessary when applied in different marine areas, increasing the complexity of typhoon wave estimation and reducing computational efficiency.

Data-driven models involve learning from experience the single or multiple influencing parameters to obtain the prediction results of typhoon waves at the study location of interest. It contains various branches, but artificial neural networks have been the most popular method in the past 20 years, mimicking the human brain in learning complex rules from abundant data [17]. Such methods overcome the limitations of regression empirical models in describing nonlinear data and, due to not requiring detailed spatial information, offer computational benefits over traditional hydrodynamic wave models, providing them an alternative method for typhoon wave prediction [12,13,18,19,20]. Although artificial neural network models have these advantages, their static network structure is limited in handling time-dependent data. The practice process of the network model in each data point is independent, meaning that the network parameters are reset after processing each data point [21,22]. This presents challenges in accurately representing time-dependent atmospheric and oceanic conditions, such as typhoon waves [23,24,25,26].

Deep learning methods have been rapidly developed in recent years, among which recurrent neural networks (RNN) with circular structures can capture the relevance of sequential information in oceanic and atmospheric conditions prediction (such as significant wave height) better than traditional artificial neural networks (ANNs) results [27,28]. Long Short-Term Memory (LSTM) is a method that adds two additional memory gate cells to the original RNN structure, handling the issue of forgetting previous information after long-term learning in RNN [29,30]. This method allows researchers to improve time series prediction results in hydraulic and oceanic engineering [27,31,32,33].

Previous research has identified two challenges in data-driven models: (1) the selection of input parameters and (2) the limited prediction lead time for predictions. Building upon the authors’ experience in typhoon-induced surge prediction models [34], this study carefully selects typhoon parameters as inputs. It utilizes LSTM to establish an accurate and long lead time typhoon wave prediction model. By leveraging the time-dependent structure of LSTM, this approach aims to improve the accuracy of typhoon-induced wave prediction and extend the prediction lead time. The results of the comparison between the backpropagation neural network (BPNN) and the LSTM methods to evaluate the effectiveness of the LSTM method in prediction improvement will be presented in the subsequent sections. The study area and data collection will be described in Section 2. Section 3 will introduce the structures of LSTM and BPNN. A comparison of the prediction results between deep learning methods and backpropagation neural network methods, along with the improvement performance, will be presented in Section 4. Finally, conclusions will be presented in Section 5.

## 2. Study Site and Data Collection

Taiwan’s government proposed the policy of ‘Nuclear-Free’ in September 2016, vigorously promoting energy transition. It plans to install over 1000 offshore wind turbines on the western coasts of Taiwan (e.g., Hsinchu and Changhua counties). However, it faces a crucial challenge from natural disasters, particularly typhoons. Owing to their propagation track in the northwest Pacific, numerous typhoons would affect or even invade Taiwan every summer and autumn and may cause severe damage to people and property. When typhoons approach the coastline, nearshore waves are affected by their intense wind shear stress and strengthened by the shallowing of the topography, leading to enormous waves being generated. This poses a severe threat to offshore wind turbines, as evidenced by the damage caused during Typhoon Soudelor in 2015, which resulted in the destruction of six turbines and a loss of NTD 7.8 billion.

**Figure 1:**
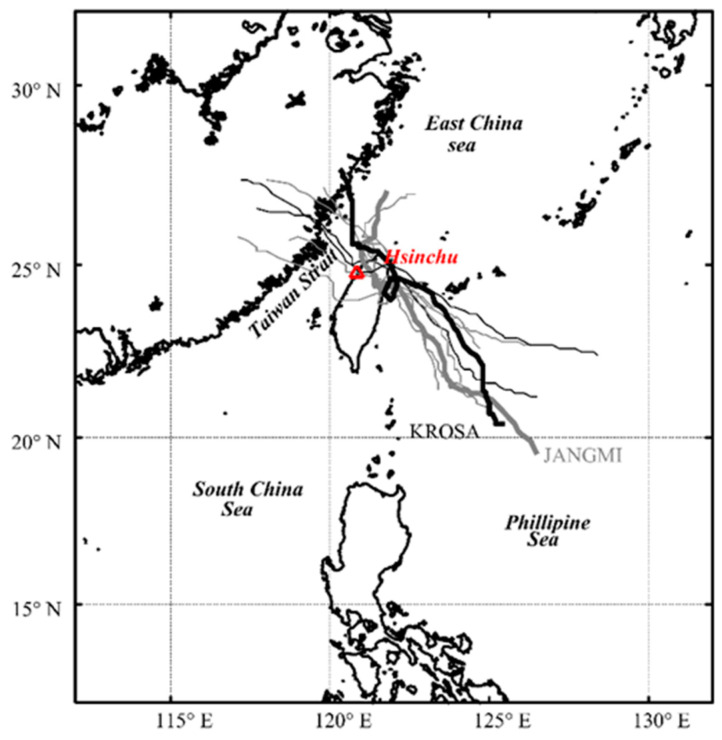
The tracks of selected historical typhoons (2006–2017) in Hsinchu station.

In recent years, the Zhunan offshore area has been selected as a site for offshore wind power development. Therefore, this study chose the nearby Hsinchu station as the research area. Typhoon information was collected from the Central Weather Administration (CWA) database from 2006 to 2017. Among the typhoon paths that most significantly affect the Hsinchu area is No. 2, as shown in Figure 1. This study utilized nine typhoon events (detailed in Table 1). The collected data revealed several severe extreme wave events, defined as significant wave heights exceeding 6 m. The most noteworthy was during Typhoon Jangmi in 2008, with a maximum significant wave height reaching 12.45 m. Table 1 lists the characteristics of all typhoon events, including minimum central pressure (*P_c_*), 10-min maximum average wind speed (*V_c_*), typhoon radius (*R_7_*), and maximum significant wave height (*H_s_*). The parameters were collected from the CWA typhoon warning report. Moreover, it also provided the latitude and longitude information of the typhoon center, and the distance (*L*) and relative angle (*θ_c_*) could be determined by the before-and-after moment (i.e., the interval time step is one hour). Finally, the forward speed (*U_F_*) and angle (*θ_F_*) were also calculated.

**Figure 2:**
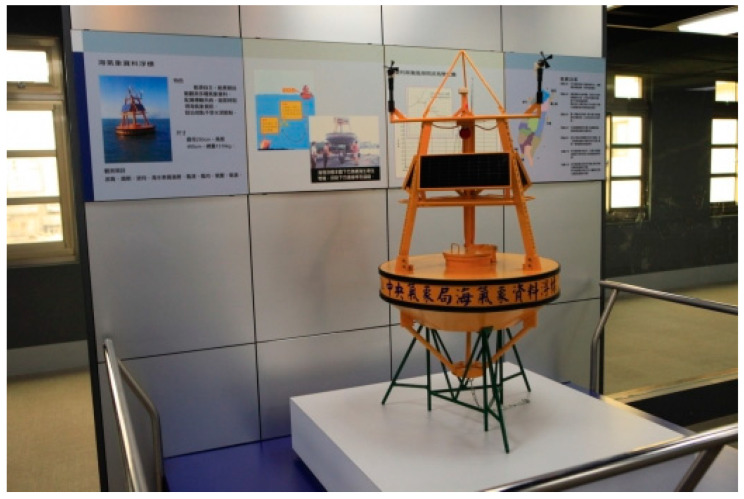
Oceanographic data buoy of Central Weather Administration, Taiwan (source: Central Weather Administration, Taiwan).

The wave data mentioned above were collected by marine meteorological data buoys established by the CWA. These buoys rely on observation techniques and data quality control from NOAA to create a localized observation system in Taiwan [35,36]. With a diameter of approximately 2.5 m (as shown in Figure 2), each buoy is equipped with a TRIAXYS wave sensor from the TRIAXYS company and a helical anemometer for wind measurement. The main parameters observed include waves, wind, air temperature, air pressure, and sea surface temperature. The wave parameters were calculated from the observed raw data from the Accelerometer–Tilt–Compass (ATC) sensors. The specifications and analysis techniques of the wave sensors were presented by Dong et al. and Lin et al. [37,38]. Power is provided by solar panels stored in batteries, and data are primarily transmitted in real time via wireless radio, GSM, GPRS, and satellite equipment.

## 3. Long Short-Term Memory and Backward Propagation Neural Network

The driving force of typhoon waves mainly comes from the wind shear stress and central pressure. Effective input parameter selection is crucial for typhoon wave prediction. Earlier studies have used the maximum wind speed of the typhoon and the angle between the typhoon and the data buoy as training factors for artificial neural networks, yielding results with prediction lead times of 1 to 3 h [12]. However, with increasing prediction lead time, more effective factors are considered to describe the correlation between the typhoon center and the data buoy to avoid reducing the training effectiveness.

In prior research, the author attempted to use the typhoon’s central pressure and maximum wind speed as driving forces and also employed the distance and relative angle between the typhoon center and the data buoy to describe their relationship. Additionally, the forward speed and angle of the typhoon, as well as the radius of maximum winds, were used to present the changes in the typhoon’s impact on the data buoy at each moment. These factors were used as input parameters, resulting in a good performance in long lead time prediction (*t* + 12 h) of typhoon storm surges [34]. However, compared to storm surges, the process of generating typhoon waves is more stochastic due to weather influences, posing a more significant challenge for training neural network systems [18,39]. It implies that it may be more challenging for traditional static network models (such as BPNN) to describe this process effectively [40]. In addition, the topography and friction disrupt the eyewall and structure of typhoons as they propagate across the Central Mountain Range (CMR), causing the lower-level center of the typhoon to weaken gradually. This study attempts to improve typhoon wave prediction results using LSTM.

The following sections will introduce the network structures of BPNN and LSTM, two data-driven modeling approaches.

### 3.1. Long Short-Term Memory Method

**Figure 3:**
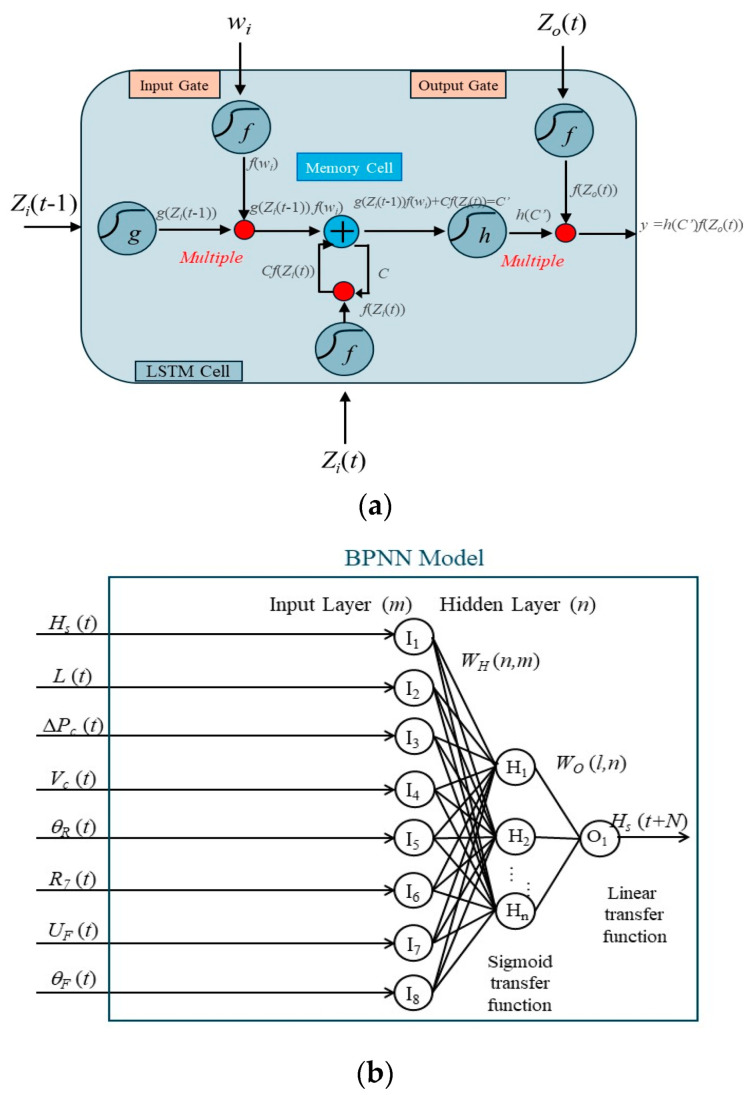
Artificial intelligence methods’ structure. (**a**) Backpropagation Neural Network; (**b**) Long Short-Term Memory.

Long Short-Term Memory is a type of recurrent neural network (RNN) that includes three gates controlling the network’s learning content: the input gate, output gate, and forget gate, as depicted in Figure 3a. The forget gate primarily determines data retention or forgetting by setting a threshold value. As recurrent networks have only one hidden state, they suffer from severe problems of vanishing gradients and exploding gradients. LSTM addresses this issue by adding a cell state structure to the recurrent network, allowing long-term data retention. This feature highlights LSTM’s powerful memory capacity, improving prediction accuracy when dealing with large amounts of oceanic and atmospheric data [41].

First, the memory cell stores an initial value called *C*. Upon inputting new data *Z_i_*(*t* − 1), the latest value *g*(*Z_i_*(*t*)) can be obtained through multiplication with the hyperbolic activation function. The flow of new information into the memory cell is controlled by the input gate. Subsequently processed by the input gate, the latest data (*C*′) can be expressed as follows:

(1) $$C^{\prime }=g\left(Z_{i}\left(t-1\right)\right)f\left(w_{i}\right)$$

where *w_i_* controls whether the gate is open, and *f(x)* is the sigmoid activation function. When *f(w_i_)* = 1, the memory cell is updated to 0. Otherwise, no update occurs.

The forget gate, implemented as a sigmoid layer, determines which information in the memory cell state should be retained or discarded. It considers the previous cell state (*C*) and the current input *Z_i_*(*t*) to generate a forget gate output between 0 and 1 for each component of the cell state. If *C_f_(Z_i_*(*t*)) equals 1, then *C* is maintained; otherwise, it is neglected. Subsequently, the renewed memory cell state is presented as follows:

(2) $$C^{\prime }=g\left(Z_{i}\left(t-1\right)\right)f\left(w_{i}\right)+Cf\left(Z_{i}\left(t\right)\right)$$

Following that, *C*′ is kept in the memory cell and marked as *C*. Before the output gate processing, *C*′ is subjected to multiplication by the hyperbolic tangent function (*tanh*(*x*)) to yield *tanh*(*C*′). The next hidden state is determined through the output gate, i.e., the filtered version memory cell state. It is shared with the next time step. And it also incorporates the previous cell state, the present *Z_o_*(*t*), and candidate cell state *C*′ to yield a value between 0 and 1. Upon processing by the output gate, the output value y can be expressed as follows:

(3) $$y=h\left(C\prime \right)f\left(Z_{o}\left(t\right)\right)$$

The study utilized a powerful programming, MATLAB R2018a, to construct the prediction model using deep learning structures. The model employed the Adam optimizer with a batch size of 27 and 235 iterations. The initial parameters, such as the maximum gradient and dropout rates, were set to 1 and 0.0055 to avoid gradient explosion and overfitting. Detailed LSTM model parameters are listed in Table 2.

### 3.2. Back Propagation Neural Network

Backpropagation neural networks have been widely used in shallow learning networks over the past two decades, consisting of input, hidden, and output layers (see Figure 3b). By defining the maximum and minimum values of input data as +1 and −1, all input data can be normalized within this range. Each hidden or output layer receives a weighted sum of inputs from the previous layer, which is then transformed into temporary or final output signals through activation functions. This study used a hidden layer with 12 neurons to balance the prediction results and avoid overfitting.

(4) $$H_{n}=f\left(w_{H_{n,m}}\right)\cdot I_{m}+B_{H_{n}}\;or\;O_{l}=f\left(w_{O_{l,n}}\right)\cdot H_{n}+B_{O_{l}}$$

where *I_m_* represents the normalized input for neuron m, *H_n_* is the temporary signal of neuron *n*, and *O_l_* is the final output signal of neuron *l*. $w_{H_{n,m}}$, $w_{O_{l,n}}$, $B_{H_{n}}$, and $B_{O_{l}}$ represent the weight and bias matrices of neurons in the hidden and output layers, respectively. They utilize the hyperbolic tangential sigmoid function and linear transfer function, denoted as *f* (*x*) = [2/(1 + *e*^−2*x*^)] − 1 and *f* (*x*) = *x*.

The training process of artificial neural networks involves continuously updating the weights and biases through error backpropagation (i.e., *e_l_* = *T_l_* − *O_l_*, where *T_l_* is the target value) to minimize the cost function *C_NN_* (detailed in Equation (5)) until reaching the maximum number of iterations or meeting the accuracy requirement.

(5) $$C_{NN}=\frac{1}{P}\sum_{P=1}^{P}\sum_{=1}^{L}e_{l}^{2}\left(P\right)$$

where *P* represents the total number of inputs. The Levenberg–Marquardt learning algorithm combines the Gauss–Newton method and gradient descent approaches to achieve the fastest (2nd order) convergence [42].

Finally, the prediction results of typhoon waves are evaluated using the root mean square error (RMSE), correlation coefficient (CC), and mean absolute error (MAE).

(6) $$RMSE=\sqrt{\frac{1}{N}\sum_{i=1}^{N}{\left[{\left(H_{m}\right)}_{i}-{\left(H_{o}\right)}_{i}\right]}^{2}}$$

(7) $$CC=\frac{\sum_{i=1}^{N}\left[{\left(H_{m}\right)}_{i}-{\bar{H}}_{m}\right]\left[{\left(H_{o}\right)}_{i}-{\bar{H}}_{o}\right]}{\sqrt{\sum_{i=1}^{N}\left[{\left(H_{m}\right)}_{i}-{\bar{H}}_{m}\right]\sum_{i=1}^{N}\left[{\left(H_{o}\right)}_{i}-{\bar{H}}_{o}\right]}}$$

(8) $$MAE=\frac{1}{N}\sum_{i=1}^{N}\left|(H_{o}{)}_{i}-H_{m}\right|$$

where *N* is the total number of data points, *H_m_* and *H_o_* represent the predicted and observed values of the typhoon waves, respectively, and the overline denotes the mean value.

## 4. Results

This section presents the model constructed using Long Short-Term Memory along with eight effective typhoon parameters to predict the variation in typhoon waves at the Hsinchu data buoy in northwestern Taiwan. It compares the results with those built using traditional static networks (i.e., BPNN). Over the past 20 years, nine typhoon events have primarily affected this area (as described in Table 1). Typhoon Krosa (2007) and Typhoon Soulik (2013) were selected as verification cases in this study for the data-driven model, which is characterized by the severe impacts of typhoon waves, while the other typhoon events are used for model training. Due to space limitations in this article, Typhoon Jangmi and Typhoon Krosa were selected as representatives for the training and validation cases, respectively. The main reason for this is that the model theoretically undergoes comprehensive training during model training by including all No. 4 typhoon path events with varying intensities from light to strong since 2006. Therefore, the two most significant wave height typhoon events were selected as representatives; if the prediction performance is good, other typhoon events can also achieve reasonable and accurate results.

The prediction lead time for typhoon waves ranges from 1 to 8 h. Section 4.1 presents the training and validation results for one prediction lead time. Due to space limitations, the following sections only present cases representing severe waves (*H_s_* > 8.0 m): Typhoon Jangmi (training) and Typhoon Krosa (verification). Section 4.2 compares the performance of LSTM and BPNN in predicting typhoon waves for long prediction lead times. Section 4.3 shows the prediction performance of each method and discusses the results.

### 4.1. One Hour Lead Time Prediction Results

**Figure 4:**
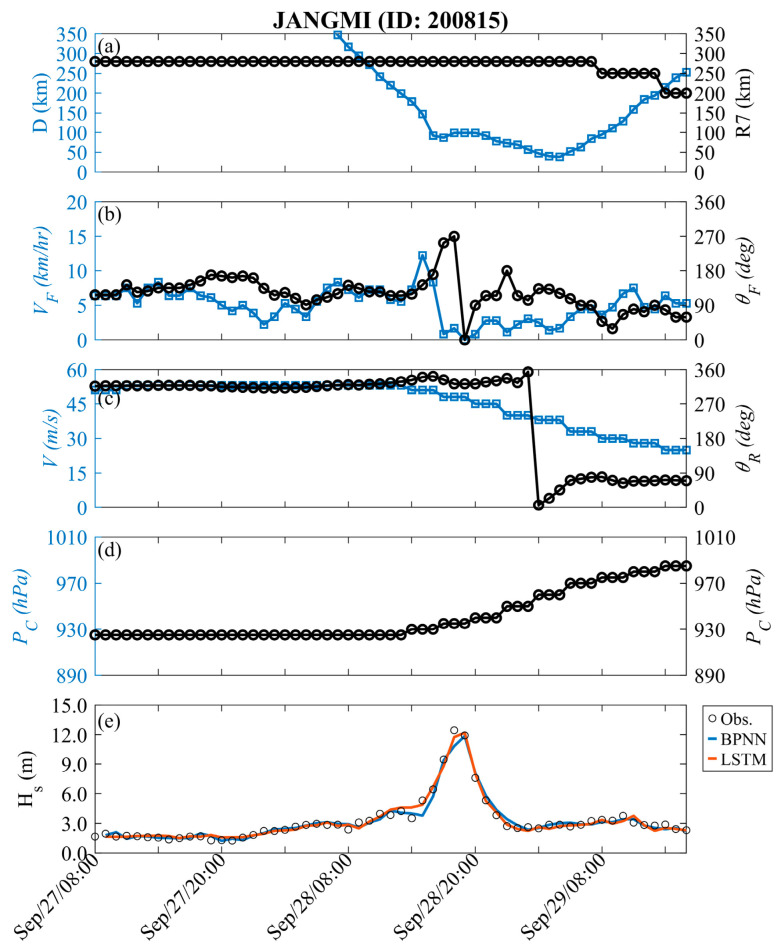
Temporal variations in typhoon parameters and *H_s_* at Hsinchu during the Jangmi event (training).

Figure 4 shows the time-series variation in typhoon factors for training events. In Figure 4a, the forward speed and angle of the typhoon are described; Figure 4b displays the relative relationship between the typhoon center and the data buoy, including distance and storm radius; Figure 4c illustrates the maximum wind speed and relative angle; Figure 4d represents the pressure deviation between the atmospheric pressure and the central of the typhoon. The variation in *H_s_* during Typhoon Jangmi is also shown in Figure 4e. At first (see Figure 1), the typhoon was located approximately 600 km southeast of the Hsinchu data buoy (i.e., at a relative angle of approximately 300°). The central pressure of Typhoon Jangmi was 925 hPa (i.e., Δ*P* = 1013 − *P_c_* = 88 hPa), with maximum wind speeds reaching 53 m/s and a storm radius of 280 km. When Typhoon Jangmi moved northwestward and approached Taiwan, its trajectory oscillated near the Central Mountain Range before turning to the northwest side (i.e., around 5:00 p.m. on 28 September 2008). During this period, the structure of Typhoon Jangmi was disrupted, leading to a decrease in the wind speed and pressure intensity at the typhoon center. When the distance between the typhoon center and the data buoy was less than the storm radius, the typhoon effects raised the wave height at the nearshore. When Typhoon Jangmi was approximately 87 km from the Hsinchu data buoy, typhoon waves exceeding 12 m were generated. Later, the typhoon waves gradually weakened. The LSTM (red line) and BPNN (blue line) methods exhibit considerable consistency with the observed values in the 1 h lead time prediction, with RMSE values of 0.343 and 0.423 m, CC values of 0.989 and 0.983, and MAE values of 0.257 and 0.291 m, respectively.

**Figure 5:**
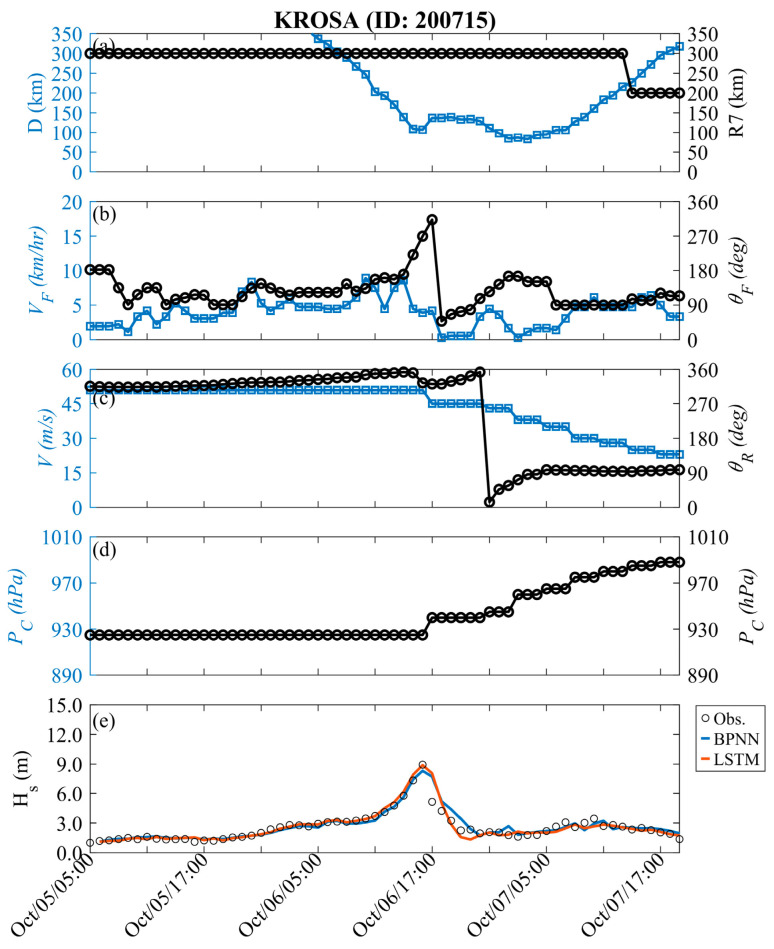
Temporal variations in typhoon parameters and *H_s_* at Hsinchu during the Krosa event (validation).

Figure 5 depicts the results of Typhoon Krosa (validation), similar to the training events. The central pressure, maximum wind speed, and storm radius of Typhoon Krosa were 925 hPa, 51 m/s, and 300 km, respectively. Unlike Typhoon Jangmi, Typhoon Krosa had a similar intensity but generated a more minor wave height (8.94 m). Due to the longer distance between the center of Typhoon Krosa and the data buoy (107 km) compared to the training events, the maximum wind speed of Typhoon Krosa was slightly higher (53 m/s). In the one hour lead time prediction, both well-trained dynamic neural network models (LSTM) and traditional static neural network methods (BPNN) demonstrated an excellent predictive performance, with RMSE values of 0.486 m and 0.499 m, CC values of 0.959 and 0.943, and MAE values of 0.272 and 0.302 m, respectively.

### 4.2. Long Lead Time Prediction Results

**Figure 6:**
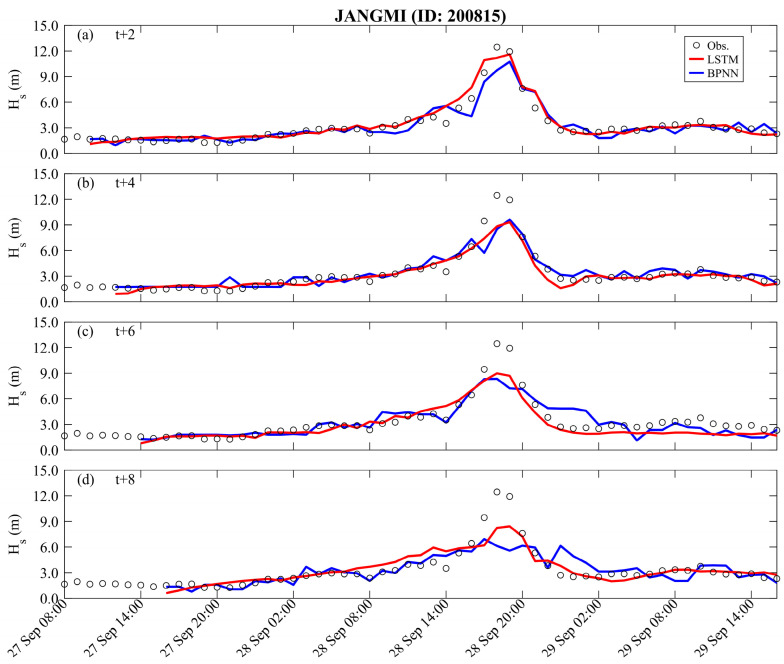
Long lead time typhoon wave predictions obtained by different methods for the Jangmi event (training): (**a**) *t* + 2; (**b**) *t* + 4; (**c**) *t* + 6; (**d**) *t* + 8.

**Figure 7:**
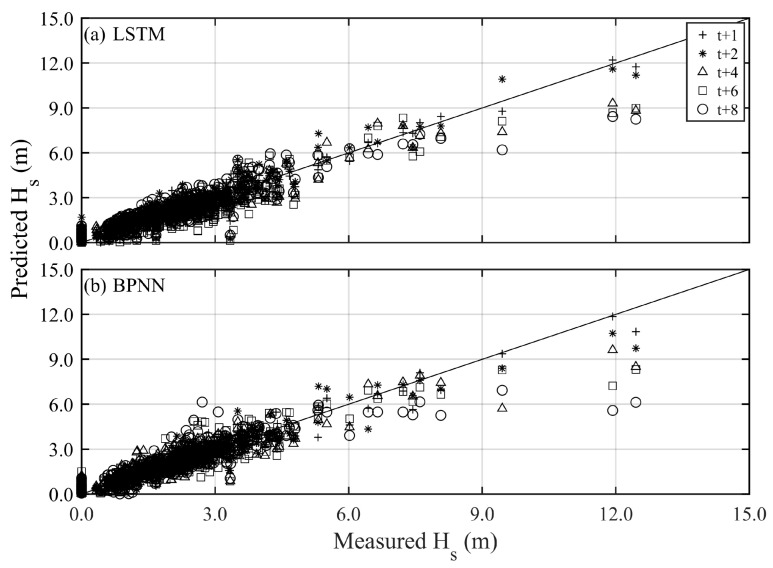
Scatter plots of predicted and measured typhoon waves for all training events under different methods and lead times (various symbols): (**a**) LSTM and (**b**) BPNN.

Figure 6 and Figure 7 present the prediction results and corresponding performance index of typhoon waves during Typhoon Jangmi (training) with long lead times (*t* + 2 to *t* + 8), respectively. The prediction results of the BPNN method can capture the variations at *t* + 2 h (see the blue line in Figure 6), with the statistical index of RMSE, CC, and MAE being 0.787 m, 0.941, and 0.520 m, respectively (see Table 2). The LSTM method (red line) more accurately describes typhoon waves’ time series and peak values than the BPNN method, with RMSE, CC, and MAE being 0.603 m, 0.969, and 0.431 m, respectively. As the lead time for typhoon wave prediction increases, both the BPNN and LSTM methods are affected in estimating the peak values of typhoon waves. Remarkably, the performance of the BPNN method decreases by 10% to 30% as the prediction time increases, compared to the LSTM method (refer to Table 2). As the prediction lead time reaches *t* + 8 h, the results of the BPNN method can only roughly describe the changing trend of typhoon waves, showing a significant underestimation in peak values, with RMSE, CC, and MAE being 1.578 m, 0.742, and 0.903 m, respectively. The LSTM method performs significantly better in predicting peak values of typhoon waves than the BPNN results, with RMSE, CC, and MAE being 1.121 m, 0.742, and 0.714 m, respectively.

**Figure 8:**
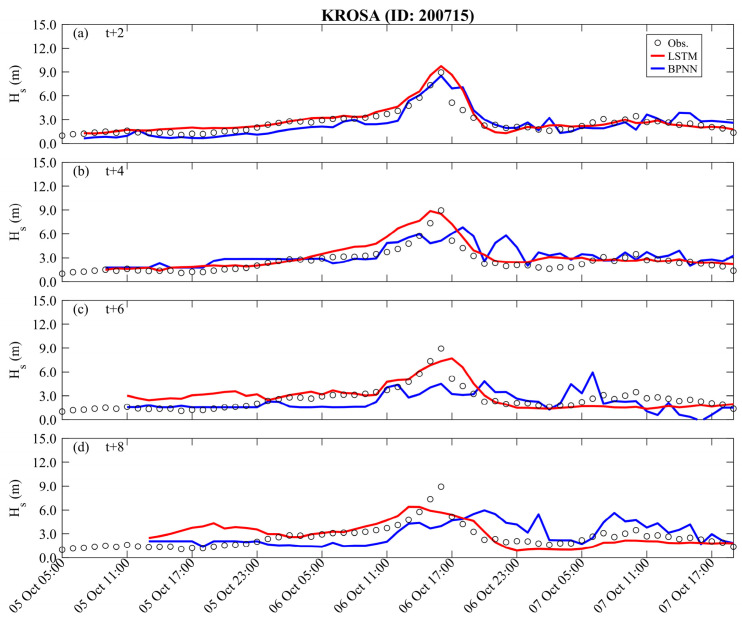
Long lead time typhoon wave predictions obtained by different methods for the Krosa event (validation): (**a**) *t* + 2; (**b**) *t* + 4; (**c**) *t* + 6; (**d**) *t* + 8.

**Figure 9:**
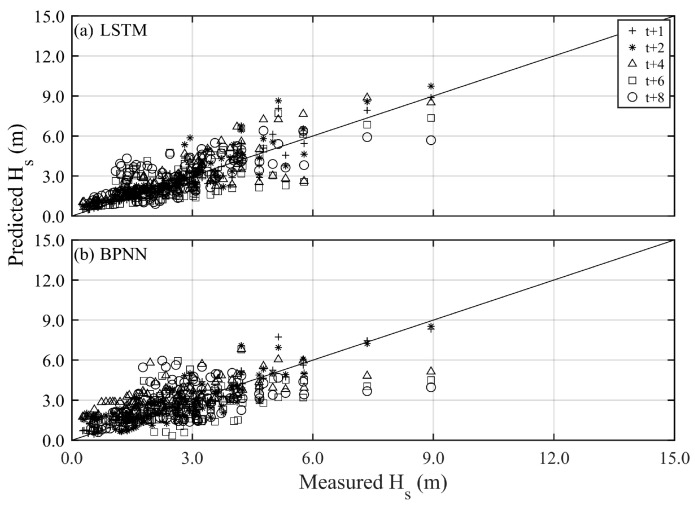
Scatter plots of predicted and measured typhoon waves for all validation events under different methods and lead times (various symbols): (**a**) LSTM and (**b**) BPNN.

The long lead time predictions for the validation results (Typhoon Krosa) are presented in Figure 8 and Figure 9. The BPNN methods can effectively describe the variations in peak typhoon waves only at *t* + 2 h, with RMSE, CC, and MAE being 0.876 m, 0.864, and 0.719 m, respectively. The performance of LSTM is slightly better than BPNN (i.e., RMSE, CC, and MAE are 0.713 m, 0.940, and 0.448 m, respectively). As the prediction lead time increases, the prediction results of the BPNN method begin to deteriorate rapidly and even fail to capture the trend of temporal variations. In contrast, the LSTM method can capture the changes in peak typhoon waves until *t* + 6 h. When the lead prediction time reaches *t* + 8 h, the BPNN’s prediction of peak typhoon waves shows a delayed phenomenon (see the blue line in Figure 8d), with RMSE, CC, and MAE being 164.45 cm, 0.325, and 1.293 m, respectively. Although the LSTM method’s ability to describe peak values starts to decline (with the statistical metrics of RMSE, CC, and MAE being 1.251 m, 0.639, and 1.001 m, respectively), it still captures the trend of impending uplift just before the peak values. (See Table 3 and Table 4).

### 4.3. Discussion on Prediction Performance Improvement

**Figure 10:**
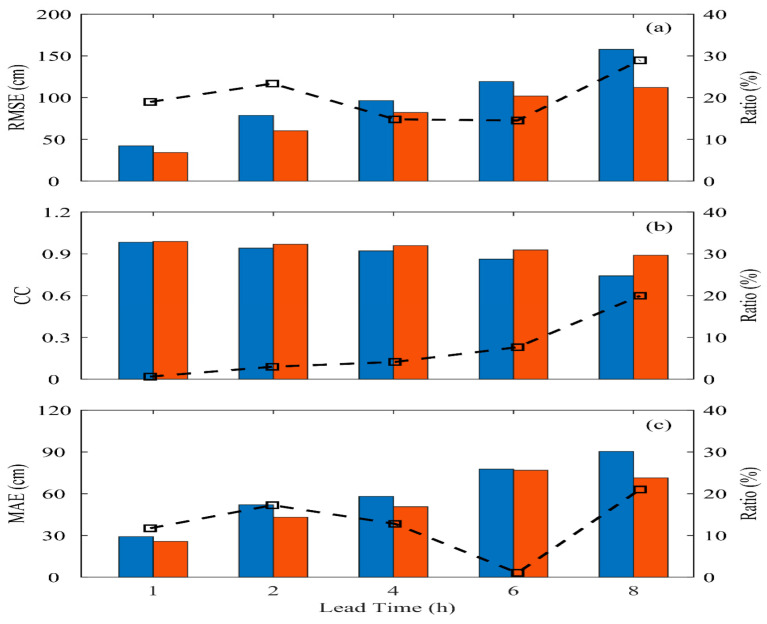
The performance (BPNN: blue bar; LSTM: red bar) and improvement (black line) of prediction in terms of (**a**) RMSE; (**b**) CC; and (**c**) MAE for all the training events.

**Figure 11:**
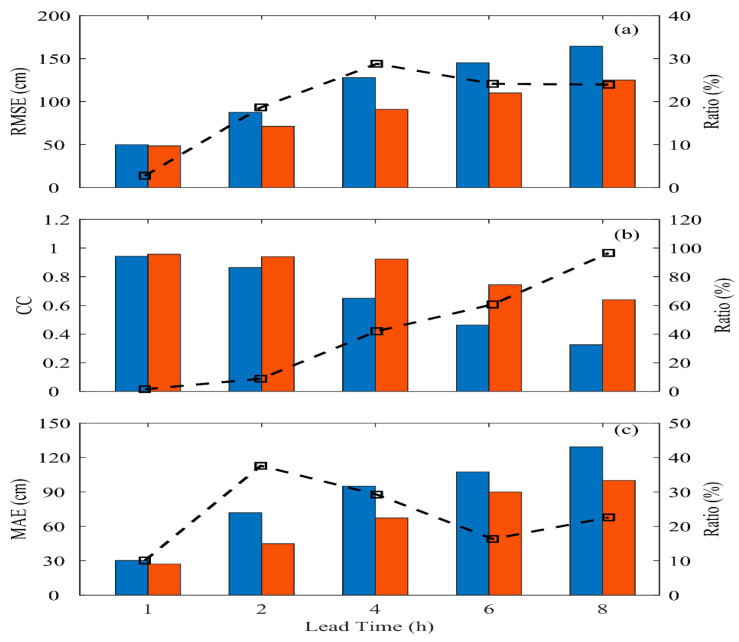
The performance (BPNN: blue; LSTM: red) and improvement (black line) of prediction in terms of (**a**) RMSE; (**b**) CC; and (**c**) MAE for all the validation events.

This section discusses the prediction performance and improvement between the LSTM and BPNN methods in the training and validation case (shown in Figure 10 and Figure 11) regarding typhoon waves. Here, the BPNN method serves as the baseline for comparison (blue bars), while the improvement magnitude of the LSTM method (red bars) compared to BPNN across different prediction lead times is examined.

In both the training and validation events, the performance of the BPNN method decreases as the prediction lead time increases. For instance, from Figure 10a, it can be observed that RMSE reduces gradually from 0.423 m to 0.903 m. Similarly, in Figure 11a, for the validation event, RMSE drops significantly from 0.499 m at *t* + 1 h to 1.645 m at *t* + 8 h. Its accuracy is generally better at short prediction lead times (within *t* + 2 h), consistent with previous research findings [34,43,44]. The prediction model established using LSTM shows notable improvements compared to the BPNN model as the prediction lead time increases. In the training and validation events, these improvements range from 15% to 29% and 2% to 29%, respectively.

In terms of the performance of the correlation coefficient (CC) (displayed in Figure 10b and Figure 11b), it is observed that LSTM’s improvement compared to BPNN is not particularly outstanding in the training event, reaching a maximum improvement of only 20% at *t* + 8 h. However, LSTM shows notable improvements in the validation event, with a maximum improvement of nearly 96% as the prediction lead time increases. This is mainly because the BPNN method can only roughly describe the temporal variations in typhoon events during training. However, during the validation event, BPNN needs to effectively reflect the information on typhoons affected by the central mountain range in predicting typhoon waves as the prediction time increases. LSTM, due to its recurrent structure, can compare previous data points and thus performs better in prediction.

Regarding the mean absolute error (MAE) coefficient performance in Figure 10c and Figure 11c, it generally shows similar results to the RMSE coefficient. Although BPNN’s performance could be better in longer prediction lead times (*t* + 4 h and above), it has successfully extended the prediction of sea conditions in northwestern Taiwan from 1 to 3 h to 4 h. The LSTM method, with its recurrent structure and memory gates controlling input from previous temporal sequences, further extends the prediction lead time to *t* + 8 h and improves the prediction results beyond BPNN. However, the crucial challenge of predicting oceanic conditions at the data buoy in western Taiwan during typhoon events is the structure of typhoon damage caused by the Central Mountain Range as typhoons move from the Pacific Ocean to the Taiwan Strait, resulting in a lower prediction accuracy compared to the data buoy in eastern Taiwan [34]. Although LSTM has dramatically improved the shortcomings of static networks, further improvements in extending the prediction lead time may require using Bidirectional Long Short-Term Memory (Bi-LSTM), which considers the influence of previous time and incorporates future influences.

## 5. Conclusions

This study utilized Long-Short-Term Memory and backpropagation neural network methods to develop a typhoon waves prediction model with a long prediction lead time of 8 h. The study area was selected at the Hsinchu Buoy Station, and nine historical typhoon events were considered for model training and validation. Regarding the prediction performance of the models, evaluations were conducted using three indices: root mean square error (RMSE), correlation coefficient (CC), and mean absolute error (MAE).

For the prediction results with a lead time of one hour, both the LSTM and BPNN methods exhibited an excellent performance in the training event (Typhoon Jangmi), with RMSE values of 0.343 m and 0.423 m, CC values of 0.989 and 0.983, and MAE values of 0.257 m and 0.291 m, respectively. Similarly, in the validation event (Typhoon Krosa), they showed a consistent prediction performance (RMSE values of 0.486 m and 0.499 m, CC values of 0.959 and 0.943, and MAE values of 0.272 m and 0.302 m for LSTM and BPNN, respectively).

In terms of long lead time prediction, BPNN was able to capture peak values in the training event at *t* + 2 h. However, the prediction performance declined rapidly as its prediction lead time increased. Particularly at *t* + 8 h, the statistical indicators for BPNN were RMSE = 1.578 m, CC = 0.742, and MAE = 0.903 m. In contrast, the LSTM method, compared to BPNN, was more accurate in describing the temporal sequence and variations in peak values. When the prediction lead time increased to *t* + 8 h, its performance was significantly better than BPNN’s (with RMSE = 1.121 m, CC = 0.742, and MAE = 0.714 m).

Regarding the improvement in model prediction, the LSTM prediction model outperformed the BPNN method in typhoon wave prediction as the prediction lead time increased to 8 h. In both training and validation events, the improvement percentages for RMSE were 15–29% and 2–29%; for CC, they were 1–20%; and for MAE, they were 1–20% and 10–40%, respectively.

Overall, this study utilized the LSTM method, which has a recurrent structure and memory gates to control the input from previous time sequences, to improve the results of shortfalls and poor prediction accuracy in the static network architecture (BPNN). However, the prediction of typhoon wave conditions in the western Taiwan data buoy is affected by the disruption of the wind field structure caused by the Central Mountain Range, resulting in a lower prediction accuracy than predictions in eastern Taiwan. This model can be effectively applied to predict significant wave heights for future events of the No. 4 typhoon path. In the future, to further increase prediction time and accuracy, the use of Bidirectional Long Short-Term Memory (Bi-LSTM), which considers both past and future influences, may be necessary for refinement. In addition, the real-time prediction model would be planned and constructed. The numerical atmospheric forecast models would be applied as input to predict the possible outcomes for the next 1 to 6 h, and once the real-time typhoon wave information is obtained, the model can be adjusted with new parameters. Finally, the real-time prediction and adjustment method would construct the early warning system.

## Figures and Tables

**Table 1:** Central pressure (*P_c_*), maximum wind speed (*V_c_*), and radius (*R_7_*) of typhoons, as well as maximum significant wave heights (*H_s_*) in historical events.

Name	Year	Path	*P_c_* (hPa)	*V_c_* (m/s)	*R_7_* (km)	Max. *H_s_* (m)
Bilis	2006	2	978	25	300	3.34
Krosa *	2007	2	925	51	300	8.94
Kalmaegi	2008	2	970	33	120	2.29
Sinlaku	2008	2	925	51	250	3.54
Jangmi	2008	2	925	53	280	12.45
Saola	2012	2	960	38	220	4.76
Soulik *	2013	2	925	51	280	5.78
Dujuan	2015	2	925	51	220	8.07
Nesat	2017	2	955	40	180	3.41

* indicates validation events.

**Table 2:** Hyperparameters of LSTM.

Hyperparameters	Value
Learning Functions	Adam
Max. Epoch	235
Min. Batch Size	27
Dropout	0.0055
Hidden Layers	10
Number of Neurons in the Hidden Layer	100

**Table 3:** Assessment of different lead time typhoon wave predictions during the Jangmi event (training).

Lead Time (h)												
	2			4			6			8		
	RMSE	CC	MAE	RMSE	CC	MAE	RMSE	CC	MAE	RMSE	CC	MAE
**LSTM**	0.603	0.969	0.431	0.822	0.959	0.506	1.020	0.928	0.768	1.121	0.890	0.714
**BPNN**	0.787	0.941	0.520	0.965	0.921	0.580	1.193	0.862	0.776	1.578	0.742	0.903

Unit: m.

**Table 4:** Assessment of different lead time typhoon wave predictions during the Krosa event (validation).

Lead Time (h)												
	2			4			6			8		
	RMSE	CC	MAE	RMSE	CC	MAE	RMSE	CC	MAE	RMSE	CC	MAE
**LSTM**	0.713	0.940	0.448	0.911	0.923	0.673	1.101	0.744	0.900	1.251	0.639	1.001
**BPNN**	0.876	0.864	0.719	1.279	0.650	0.951	1.452	0.463	1.075	1.645	0.325	1.293

Unit: m.

## Data Availability

Data are contained within the article.
